# Identification of Risk‐Related Prenatal Medication Exposure Profiles for Childhood Asthma Using the Bayesian Profile Regression: A Cohort Study Using the POMME Cohort

**DOI:** 10.1002/pds.70509

**Published:** 2026-10-19

**Authors:** Solène Russo, Justine Bénévent, Anna‐Belle Beau

**Affiliations:** ^1^ CERPOP, Inserm U1295 Toulouse France; ^2^ CERPOP, Inserm U1295, Université de Toulouse, Service de Pharmacologie Clinique, CHU de Toulouse Toulouse France

**Keywords:** asthma, Bayesian profile regression, prenatal drug exposure

## Abstract

**Purpose:**

To identify prenatal medication exposure profiles associated with childhood asthma.

**Methods:**

This population‐based cohort study used data from the POMME (PrescriptiOn Médicaments Mères Enfants) cohort, including 18 851 children born in the Haute‐Garonne area (France) in 2010 and 2015, and followed until 2023. Prenatal exposure was defined as medications dispensed to mothers during pregnancy, classified by Anatomical Therapeutic Chemical subclasses, assessed separately for each trimester as none, one dispensing, or at least two dispensings. Childhood asthma from Age 4 years onward was identified using an algorithm based on dispensing of asthma medications. Bayesian profile regression was applied to simultaneously cluster children according to prenatal medication exposure profiles and asthma occurrence. High‐risk profiles were characterised by predominant medication classes, timing of exposure and maternal and infant characteristics.

**Results:**

Seventeen prenatal medication exposure profiles were identified. Six profiles were associated with a higher average risk of childhood asthma compared with the overall population risk (15.2%). High‐risk profiles were characterised by repeated exposure throughout pregnancy to medications for acid‐related disorders, thyroid hormones, antibiotics, asthma medications and antihistamines, as well as by combinations involving antibiotics, nasal medications and cold medicines in late pregnancy. Mothers in high‐risk profiles more frequently presented chronic conditions and pregnancy‐related complications.

**Conclusion:**

This exploratory study identified prenatal medication exposure profiles associated with childhood asthma using a semi‐supervised Bayesian approach. This method highlights combinations and critical windows that may warrant further investigation and supports Bayesian profile regression as a useful tool for signal detection in perinatal pharmacoepidemiology.

## Key Points

- Previous studies have reported associations between prenatal exposure to specific medication classes, such as antibiotics or acid‐suppressive drugs and childhood asthma, but have generally evaluated medications individually.
- Medication use during pregnancy commonly involves co‐medication and varying exposure timing, which are insufficiently captured by traditional analytical approaches.
- Using a semi‐supervised Bayesian approach, this study identified distinct prenatal medication exposure profiles characterised by combinations of therapeutic classes and timing of exposure.
- Several exposure profiles were associated with higher average risks of childhood asthma, highlighting the potential role of exposure patterns rather than single medications.
- Data‐driven exposure profiling may support signal detection in perinatal pharmacoepidemiology and help prioritise medication combinations and exposure windows for future causal research.

## Plain Language Summary

Asthma is one of the most common chronic diseases in children. While genetics and environmental factors such as air pollution are known to play a role, researchers are increasingly interested in whether exposures during pregnancy may also influence a child's future risk of asthma. Many pregnant women use medications, often more than one at a time, but most previous studies have examined single medications separately rather than considering combinations of drugs or the timing of their use during pregnancy. In this study, we analysed data from nearly 19 000 children born in France and followed from birth into childhood. We used a statistical method that groups children according to similar patterns of maternal medication use during pregnancy. This approach allowed us to identify different ‘exposure profiles’ based on the types of medications used and when they were taken. We found several distinct medication exposure profiles, some of which were associated with a higher average risk of childhood asthma. These results highlight specific combinations of medications and periods during pregnancy that may deserve further investigation to better understand medication safety during pregnancy.

## 1 Introduction

Pregnancy is a high‐risk period during which exposure to medication can affect the short‐ and long‐term health of the child. In France, more than 90% of pregnant women receive at least one dispensing [1], and many medications cross the placenta, directly exposing the foetus. While the short‐term effects of certain medications are well documented, their potential long‐term consequences on child health, particularly on respiratory outcomes, remain poorly understood.

Childhood asthma, which affects 10%–16% of children in France [2], is one of the most common chronic diseases in paediatric populations. Its aetiology is multifactorial, involving genetic susceptibility and environmental factors such as tobacco smoke and air pollution, but also potentially prenatal exposures. Increasing evidence suggests that prenatal medication exposure may influence immune and respiratory development and increase susceptibility to asthma later in childhood. Several studies have reported associations between specific medication classes, including antacids [3] and antibiotics [4] and childhood asthma risk, although findings remain inconsistent and partly explained by familial or maternal confounding [5, 6]. Most previous studies focused on single medication classes and did not account for the complexity of medication use during pregnancy, which is characterised by co‐medications, repeated dispensings and varying exposure windows [7].

Addressing these limitations requires analytical approaches capable of jointly modelling multiple correlated exposures. Bayesian profile regression (BPR) offers a powerful data‐driven framework for this purpose, allowing individuals to be clustered according to multidimensional exposure profiles while simultaneously modelling their association with a health outcome.

The objective of this study was to apply BPR to identify risk‐related prenatal medication exposure profiles associated with childhood asthma. This exploratory, hypothesis‐generating approach aims to detect potential signals involving individual medications, combinations of medications, or specific exposure windows that may be relevant to the development of childhood asthma.

## 2 Method

### 2.1 Data Source

This population‐based cohort study draws on the French POMME (PrescriptiOn Médicaments Mères Enfants) cohort [8]. The cohort includes children covered by the general health insurance scheme who were born in the Haute‐Garonne area (France) between 1 July 2010 and 30 June 2011, and between 1 July 2015 and 30 June 2016.

POMME contains anonymised data from three routinely collected data sources:

− the Haute‐Garonne French Health Insurance Database, providing data on medicines prescribed, dispensed and reimbursed to the mothers (from two and a half months before the start of pregnancy until childbirth) and to the child. It also includes data on medical care to children from birth.
− the Mother and Child Protection Centre Database, providing data from mandatory health certificates completed by doctors during medical examinations of children at birth, at 9 months and at 24 months.
− and the Toulouse University Hospital, providing hospitalisation data (dates of in‐patient stay and diagnoses recorded according to CIM‐10) for mothers during pregnancy.

The record linkage was performed deterministically using the mother's married and maiden names, first name of the mother and the child's date of birth, achieving an overall linkage rate of around 80%.

### 2.2 Study Population

The POMME cohort included 18 851 children which were followed until the end of 2023 (the last year of available data) or until the last date of any reimbursement, whatever came first. Consequently, follow‐up extended up to a maximum of 12 years for children born in 2010–2011 and 7 years for those born in 2015–2016.

### 2.3 Exclusion Criteria

Several exclusion criteria were applied (Figure 1). Children with less than 4 years follow‐up (defined as no reimbursed healthcare use from birth to age 4 years) were excluded, as asthma cannot be reliably diagnosed before this age. Very preterm babies (< 30 weeks of gestation) were excluded due to their high risk of respiratory complications unrelated to asthma. Children with cystic fibrosis or major congenital respiratory anomalies according to the EUROCAT classification [9], (ICD‐10 codes: Q30.0, Q32.0, Q33.0 (excluding Q33.6), Q34.0) were also excluded. Children without prenatal medication exposure were excluded, as their exposure profile would consist entirely of zero values and would not provide meaningful information for the identification of distinct prenatal medication exposure patterns. Children whose mothers were exposed only to medication subclasses or active ingredients widely available over‐the‐counter or generally considered safe during pregnancy (including vasoprotectors: C05, homeopathy medications, antianaemic agents: B03, vitamins: A11, paracetamol: N02BE01, phloroglucinol: A03AX12, heparin sodium: B01AB01) were excluded.

**FIGURE 1:**
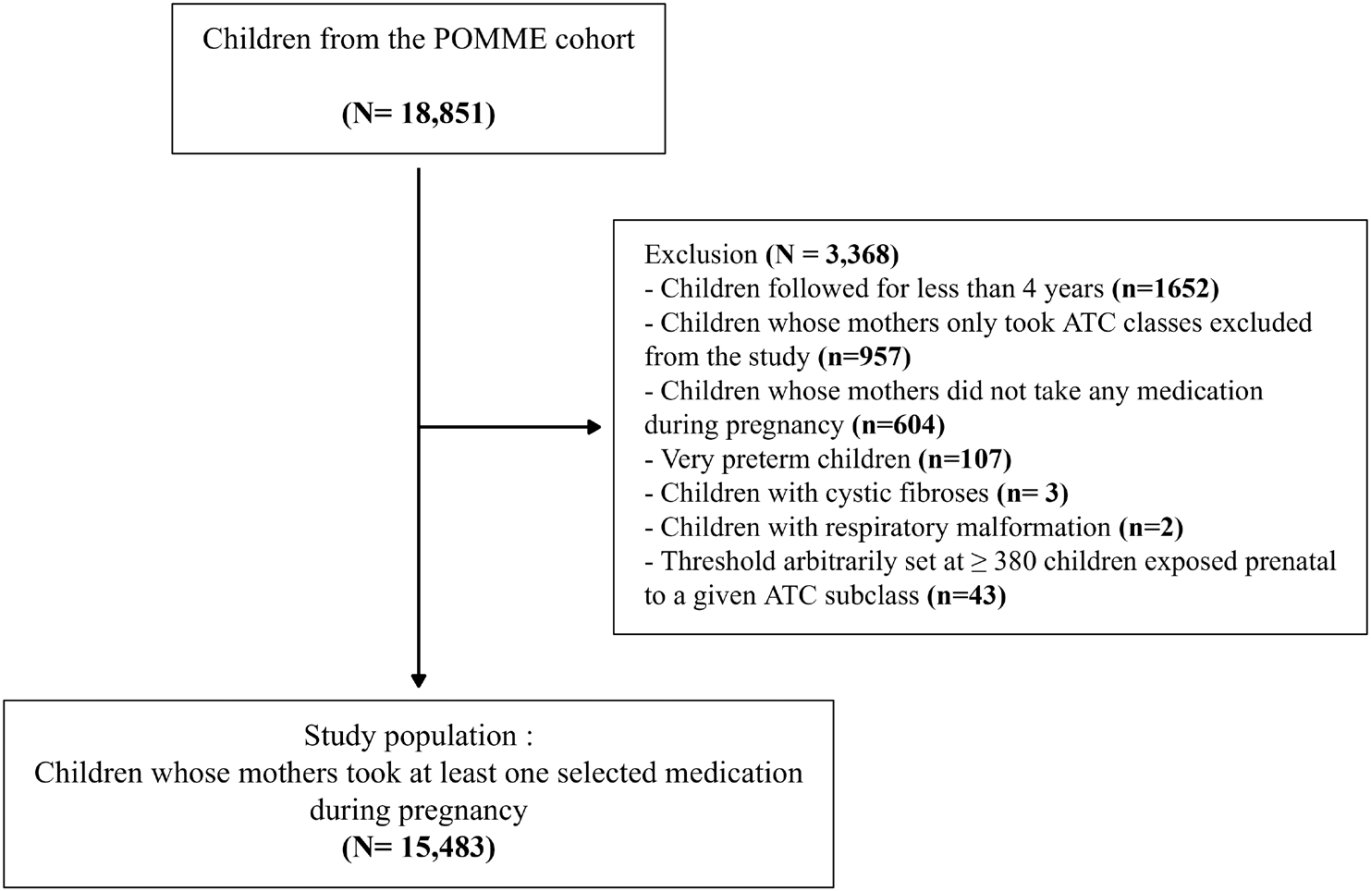
Flowchart describing the study population selection.

### 2.4 Exposure Definition

Prenatal medication exposure was defined as any medication dispensed to the mother during pregnancy, identified using the Anatomical Therapeutic Chemical (ATC) Level 2 classification [10]. To limit the number of sparse exposure variables included in the clustering analysis, only medication exposures recorded in at least 380 pregnancies (< 2% of the POMME cohort) were considered, resulting in 36 medication subclasses (Table S1). This pragmatic threshold was chosen to balance the inclusion of informative medication exposures with model complexity and the stability and interpretability of the resulting exposure profiles. Exposure was assessed separately for each trimester of pregnancy [11]: first (0–13 weeks of amenorrhea), second (14–27 weeks) and third (≥ 28 weeks until delivery). For each ATC subclass and trimester, exposure was categorised into three mutually exclusive categories: no exposure, one dispensing, or at least two dispensings during the corresponding period.

### 2.5 Outcome Definition

The primary outcome was childhood asthma occurring from age 4 years onwards. As diagnostic data were not available in the cohort, asthma was identified using an algorithm based on dispensing records [12], for both rescue and maintenance medications [13, 14] (Table S2). A child was classified as having asthma if he received at least three dispensings of rescue medications during follow‐up, or at least three consecutive dispensings of maintenance therapy with a maximum interval of 42 days between dispensings, corresponding to standard treatment durations of 28‐day with a 14‐day grace period [15].

### 2.6 Statistical Analysis

Bayesian profile regression was used to identify clusters of children with similar prenatal medications exposure profiles and to estimate their association with childhood asthma. This semi‐supervised Bayesian approach jointly incorporates multidimensional exposure variables (number of dispensings for each medication subclass by trimester) and the binary outcome into the cluster formation process, generating groups that are homogeneous in terms of exposure while differing in asthma risk. The model estimation is performed using Markov chain Monte Carlo sampling, generating multiple alternative partitions that were summarised through model averaging across the posterior distribution to account for uncertainty [16]. For each cluster, the average risk of asthma and corresponding 95% credibility interval (CrI) were estimated. Clusters with an average asthma risk higher than that of the total population were defined as high‐risk clusters, while remaining clusters formed the reference group.

To identify exposure variables most influential in cluster formation, differences in posterior exposure probabilities between each cluster and the reference group were calculated for each ATC subclass–trimester combination. These differences were scaled using min–max normalisation within clusters to facilitate comparison and graphical representation for the ‘at least two dispensings’ modality (Figure 2) and the ‘one dispensing’ modality (Table S3). Figure 2 focuses on the positive part of the scale (values > 0), which represents a higher prevalence of the corresponding medication exposures within a given profile. The complete figure with scale from [−1;1] is presented in Figure S4.

**FIGURE 2:**
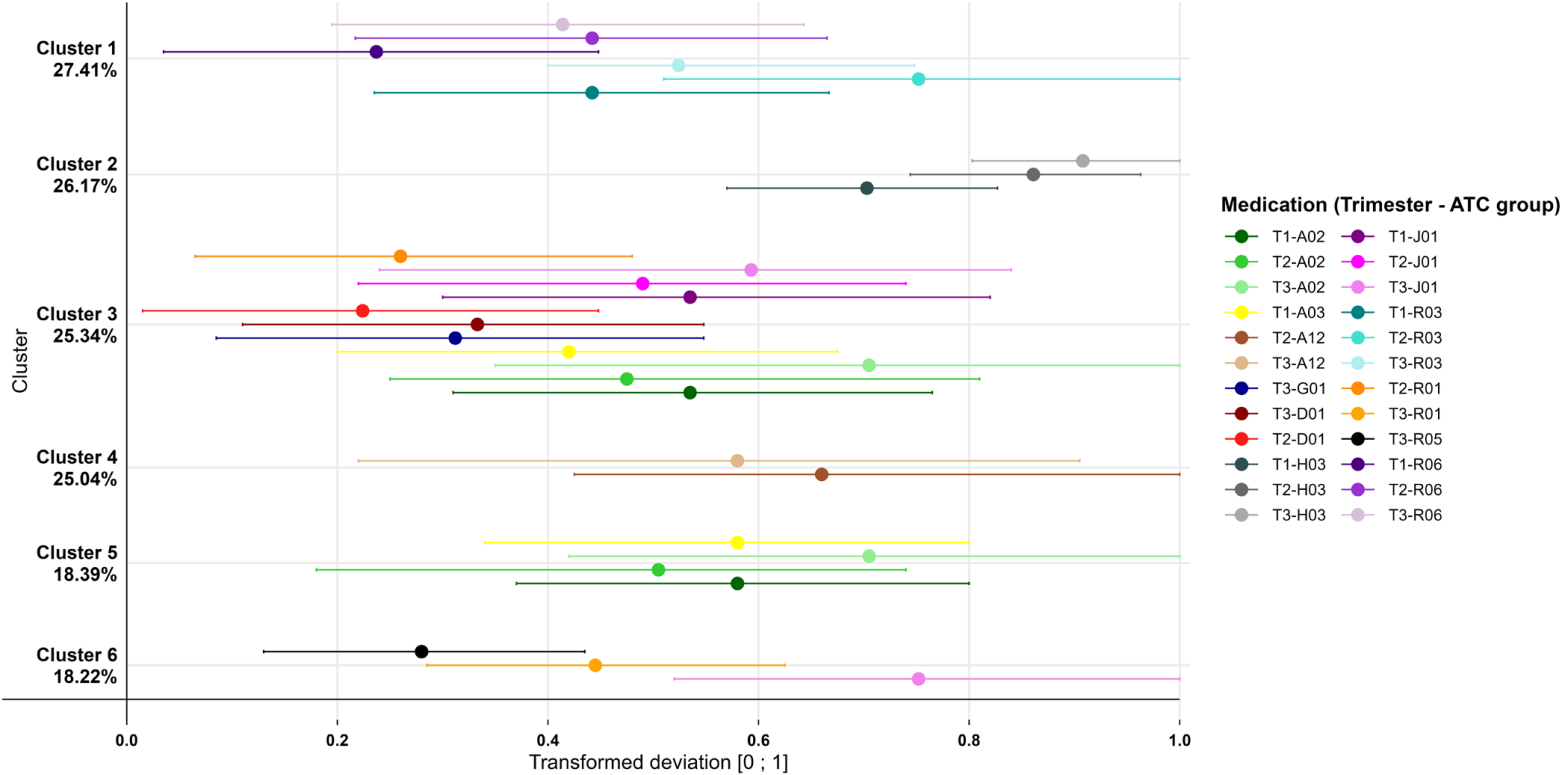
Transformed difference in average exposure probabilities (at least two dispensings) in the 6 high‐risk clusters. Positive associations (values > 0), indicating that the prevalence of specific medication exposures was higher within the profiles than in the reference group, defined as clusters with a prevalence below the population average.

Maternal and infant characteristics were described and compared between high‐risk clusters and the reference group. Quantitative variables were summarised using means (and standard deviations) and qualitative variables using frequencies (and percentages). Group comparisons were performed using Chi‐square or Fisher's exact tests (if the sample size was less than 5 cases) for categorical variables and Welch's test for continuous variables, with a two‐sided significance level of 5%.

Missing data on maternal and infant characteristics were described. No missing data were present for exposure or outcome variables. Clustering analyses were performed using the PReMiuM package [17], while data visualisation was performed using the ggplot2 package. Statistical analyses and data manipulation were carried out using the stats and dplyr packages. All analyses were performed with R software (version 4.3.2).

## 3 Results

Among the 18 851 children included in the POMME cohort, 15 483 met the inclusion criteria and were retained for the analyses (Figure 1).

### 3.1 Identification of Medication Exposure Profiles

The BPR identified 17 prenatal medication exposure profiles, grouping children according to similarities in maternal medication dispensing patterns across pregnancy and their association with childhood asthma occurrence. Table 1 presents the size of each cluster and the estimated average risk of asthma in the total population and within each cluster. The average risk of childhood asthma in the overall study population was estimated at 15.2% (95% CrI: [14.6%; 15.7%]). Six clusters exhibited an average asthma risk higher than that of the total population, with credibility intervals that did not overlap with the population‐level estimate. These clusters were therefore defined as high‐risk profiles. Cluster 1 showed the highest estimated risk of childhood asthma (27.4%, 95% CrI: [23.6; 31.2]), followed by clusters 2 (26.2%, 95% CrI: [20.7; 32.1]), Cluster 3 (25.3%, 95% CrI: [21.9; 28.9]) and Cluster 4 (25.0%, 95% CrI: [17.8; 33.2]). Clusters 5 and 6 showed intermediate but still elevated risks (ranging from 20% to 18%). The remaining 11 clusters had asthma risks similar to or lower than that observed in the total population and were grouped into a reference group for comparisons.

**TABLE 1:** Estimated average risk of asthma according to cluster assignment (BPR method).

Cluster		No of children	Estimated average risk of asthma [95% CI]
	Total population	15 483	15.17% [14.60; 15.72]
	1	364	27.41% [23.63; 31.15]
	2	188	26.17% [20.68; 32.13]
	3	483	25.34% [21.89; 28.87]
	4	99	25.04% [17.84; 33.18]
	5	677	18.39% [16.22; 20.69]
	6	1019	18.22% [16.28; 20.39]
Reference group	7	1586	16.44% [14.87; 17.96]
	8	565	16.05% [13.26; 18.79]
	9	1158	15.93% [14.27; 17.62]
	10	325	15.90% [12.79; 19.07]
	11	786	15.18% [13.09; 17.45]
	12	126	14.87% [9.69; 20.72]
	13	858	13.40% [11.33; 15.85]
	14	808	13.39% [11.27; 15.67]
	15	99	13.12% [8.19; 19.80]
	16	212	12.42% [8.56; 17.09]
	17	6130	12.31% [11.57; 13.12]

### 3.2 Characterisation of High‐Risk Exposure Profiles

The exposure variables that contributed the most to the formation of the six high‐risk clusters are presented in Figure 2 and Table S3 and Figure S4. Table 2 presents the characteristics of the children and mothers in each cluster compared with those from children in the reference group.

**TABLE 2:** Description of maternal and infant characteristics in the 6 high‐risk clusters compared individually with the reference group (weighted average of the remaining clusters with a lower than average or equal risk of asthma than the total population).

	Cluster 1	Cluster 2	Cluster 3	Cluster 4	Cluster 5	Cluster 6	Reference group
	*N* = 364	*N* = 188	*N* = 483	*N* = 99	*N* = 677	*N* = 1019	*N* = 12 653
Maternal characteristics: mean (± standard deviation); *n* (%)							
Maternal age (year)	31.87 (±4.9)^a^	32.85 (±5.0)^a^	30.95 (±5.4)	32.32 (±4.6)^a^	31.37 (±5.3)	30.61 (±5.0)^a^	31.0 (±4.8)
Gravidity^b^	2.36 (±1.5)^a^	2.32 (±1.5)	2.60 (±1.5)^a^	2.33 (±1.4)	2.19 (±1.3)	2.15 (±1.2)	2.09 (±1.3)
Missing data	46 (12.6)	30 (16.0)	81 (16.8)	15 (15.1)	99 (14.6)	139 (13.6)	1662 (13.1)
Parity^c^	1.87 (±1.0)^a^	1.87 (±1.0)^a^	2.08 (±1.3)^a^	1.83 (±0.9)	1.79 (±1.0)	1.76 (±0.8)	1.71 (±0.9)
Missing data	51 (14.0)	30 (16.0)	84 (17.4)	19 (19.1)	105 (15.5)	146 (14.3)	1780 (14.1)
At least one long‐term condition registered^d^	20 (5.5%)^a^	22 (11.7)^a^	26 (5.4)^a^	7 (7.1)^a^	43 (6.4)^a^	26 (2.6)	337 (2.7)
Maternal unemployment	68 (20.1)	40 (24.0)	138 (34.6)^a^	5 (5.7)^a^	141 (23.3)^a^	174 (19.2)	2186 (19.0)
Missing data	25 (6.9)	21 (11.2)	84 (17.4)	12 (12.1)	73 (10.8)	114 (11.2)	1123 (8.8)
Maternal level of education	^a^	^a^	^a^		^a^		
Primary education	4 (1.6)	7 (5.6)	17 (5.6)	1 (1.9)	9 (2.0)	10 (1.3)	120 (1.3)
Lower secondary education	42 (16.4)	15 (11.9)	78 (25.9)	6 (11.5)	75 (17.0)	110 (14.6)	1113 (12.2)
Upper secondary education	67 (26.2)	37 (29.4)	97 (32.2)	9 (17.3)	130 (29.5)	171 (22.7)	1972 (21.6)
Higher education	143 (55.9)	67 (53.2)	109 (36.2)	36 (69.2)	227 (51.5)	462 (61.4)	5879 (64.5)
Missing data	108 (29.7)	61 (32.4)	182 (37.7)	47 (47.5)	226 (33.4)	266 (26.1)	3535 (27.9)
Gestational age (in weeks of amenorrhea)	39.5 (±1.7)	39.5 (±1.4)	39.4 (±1.6)^a^	39.4 (±1.8)	39.4 (±1.6)^a^	39.7 (±1.4)^a^	39.6 (±1.6)
Multiple pregnancy	12 (3.3)	10 (5.3)	29 (6.0)^a^	9 (9.1)^a^	34 (5.0%)^a^	25 (2.5)	399 (3.2)
Smoking during pregnancy	20 (9.6)	9 (8.4)	41 (16.9)^a^	< 3	44 (11.9)	66 (11.4)	702 (9.7)
Missing data	156 (42.9)	81 (43.1)	241 (50.0)	50 (50.5)	306 (45.2)	442 (43.4)	5429 (42.9)
Preeclampsia	11 (3.2)^a^	< 3	13 (2.9)^a^	6 (6.4)^a^	9 (1.4)	11 (1.1)	175 (1.5)
Missing data	17 (4.7)	7 (3.7)	42 (8.7)	5 (5.1)	39 (5.8)	58 (5.7)	653 (5.1)
Pregnancy‐related hypertension	20 (5.5)	15 (8.0)^a^	44 (9.1)^a^	9 (9.1)^a^	48 (7.1)^a^	46 (4.5)	566 (4.5)
Diabetes	18 (5.2)	18 (9.9)	31 (7.0)	7 (7.4)	38 (6.0)	51 (5.3)	739 (6.2)
Missing data	17 (4.7)	7 (3.7)	42 (8.7)	5 (5.1)	39 (5.8)	58 (5.7)	653 (5.2)
Gestational diabetes	15 (4.1)	11 (5.9)^a^	26 (5.4)^a^	5 (5.1)	26 (3.8)^a^	19 (1.9)	333 (2.6)
Infant characteristics: *n* (%)							
Sex							
Female	183 (50.3)	91 (48.4)	254 (52.6)	46 (46.5)	318 (47.0)	511 (50.1)	6181 (48.9)
Male	181 (49.7)	97 (51.6)	229 (47.4)	53 (53.5)	359 (53.0)	508 (49.9)	6471 (51.1)
Preterm birth	22 (6.0)	5 (2.7)	30 (6.2)	10 (10.1)	52 (7.7)^a^	38 (3.7)^a^	723 (5.7)
Breastfeeding at birth	220 (67.5)	116 (68.2)	283 (68.4)	65 (69.9)	401 (67.6)	597 (65.7)^a^	7863 (69.9)
Missing data	38 (10.4)	18 (9.6)	69 (14.3)	6 (6.1)	84 (12.4)	110 (10.8)	1397 (11.0)

*Note:* Percentage and mean are calculated without missing data. < 3: cell with counts less than 3 are masked.

^a^ *p*‐value lower than 0.05% when comparing with the reference group (weighted average of clusters with low risk of asthma).

^b^ Total number of pregnancies a woman has had, regardless of outcome (including miscarriages, abortions, etc.).

^c^ Number of children born alive or viable (≥ 22 weeks of gestation).

^d^ Chronic condition recognized as serious and requiring prolonged follow‐up and costly treatment, entitling the patient to 100% coverage by health insurance (e.g., diabetes, cancer, heart failure, etc.).

Cluster 1, comprising 364 children with an estimated asthma risk of 27.4% (CrI: [23.6; 31.2]), was characterised by exposure to at least two dispensings of asthma medications (R03) and antihistamines (R06) during all three trimesters of pregnancy. Mothers in this cluster more frequently had a recorded long‐term condition and pre‐eclampsia than those in the reference group. Cluster 2, comprising 188 children, with an estimated asthma risk of 26.1% (CrI: [20.7; 32.1]), was primarily characterised by exposure to at least two dispensings of thyroid hormones (H03) throughout pregnancy. Mothers in this cluster were older and more frequently had a record of long‐term condition and pregnancy‐related hypertension than those in the reference group. Cluster 3, with 483 children and an estimated asthma risk of 25.3% (CrI: [21.9; 28.9]), included children who received at least two dispensings of antacids (A02) and antibiotics (J01) during all trimesters of pregnancy, as well as at least two dispensings of gastrointestinal medications (A03) in the first trimester and at least two dispensings of antimycotic medications (D01), anti‐infective medications (G01) and nasal medications (R01) in the second and third trimesters. In addition, a single dispensing of systemic corticosteroids (H02) was observed in the first trimester. Mothers in this group had lower levels of education, higher unemployment rates and had higher percentages of smoking during pregnancy, long‐term conditions, pregnancy‐related hypertension and gestational diabetes compared with those in the reference group. Cluster 4, comprising 99 children with an estimated asthma risk of 25.0% (CrI: [17.8; 33.2]), was characterised by exposure to at least two dispensings of mineral supplements (A12) in the second and third trimesters. Compared with the reference group, mothers were older, more frequently employed and more often had long‐term condition, preeclampsia and pregnancy‐related hypertension. Multiple births and premature delivery were more frequent in this cluster, and infants were less frequently breastfed at birth. Cluster 5, including 677 children with an estimated asthma risk of 18.4% (CrI: [16.2; 20.7]), was characterised by exposure to at least two dispensings of acid suppressant medications (A02) in all three trimesters of pregnancy and at least two dispensings of medications for gastrointestinal functional disorders (A03) during the first trimester. Mothers in this group more frequently had a lower educational level, long‐term conditions, pregnancy‐related hypertension and gestational diabetes compared with those in the reference group. Preterm birth was more frequent than in the reference group. Finally, Cluster 6, comprising 1019 children with an estimated asthma risk of 18.2% (CrI: [16.3; 20.4]), was characterised by exposure to at least two dispensings of antibiotics (J01), cold and cough medicines (R05) and nasal medicines (R01) during the third trimester of pregnancy. Infants were less frequently born preterm and were less often breastfed at birth than those in the reference group.

## 4 Discussion

In this population‐based cohort study using a data‐driven, semi‐supervised Bayesian approach, we identified 17 distinct prenatal medication exposure profiles, six of which were associated with a higher risk of childhood asthma than that observed in the overall population (15.2%). The profiles associated with the highest asthma risk involved exposure throughout pregnancy to medications for acid‐related disorders, thyroid hormones, systemic antibiotics, asthma medications and antihistamines. Other profiles were characterised by more specific combinations of medications or by exposure concentrated in late pregnancy, notably involving antibiotics, nasal preparations and cough and cold medicines.

### 4.1 Comparison With Existing Literature

Our findings are broadly consistent with previous observational studies reporting associations between prenatal exposure to certain medication classes and childhood asthma, while extending this literature by highlighting the importance of the clinical context, combinations and timing of medication exposure. Importantly, the interpretation of the six high‐risk profiles suggests that some associations may reflect potential effects of prenatal medication exposure, particularly specific combinations and timing of exposure, while also highlighting the importance of considering underlying maternal disease and treatment indication, as well as broader patterns of multimorbidity and healthcare utilisation.

The profile identified in Cluster 1, characterised by repeated exposure to asthma medications and antihistamines throughout pregnancy, was associated with the highest estimated risk of childhood asthma. However, this profile also included a higher prevalence of maternal long‐term conditions and is likely to predominantly reflect pregnancies of women with asthma and/or allergic conditions. Maternal asthma is a well‐established risk factor for asthma in offspring, and a recent meta‐analysis showed that maternal asthma significantly increases the risk of childhood asthma [18]. Thus, the association observed in this cluster is unlikely to provide evidence of a specific pharmacological effect of asthma medications or antihistamines. Rather, it illustrates the importance of considering the indication for treatment and shared familial susceptibility when interpreting associations between prenatal medication exposure and childhood asthma. A similar interpretation applies to Cluster 6, characterised by exposure to antibiotics, nasal preparations and cough and cold medicines in the third trimester. These medications may reflect maternal respiratory or allergic symptoms, and therefore the observed association may again be driven, at least in part, by the underlying maternal condition rather than by the medications themselves.

Likewise, the increased risk observed in Cluster 2, characterised predominantly by repeated exposure to thyroid hormone replacement throughout pregnancy, should be interpreted with caution. Thyroid hormone replacement is intended to correct the hormonal deficit associated with hypothyroidism and restore euthyroidism, and there is no clear biological rationale for considering replacement therapy itself as a direct risk factor for childhood asthma. In contrast, maternal thyroid disorders, particularly autoimmune thyroid disease, may reflect broader immune dysregulation or susceptibility to allergic disease. Consistent with this interpretation, maternal hypothyroidism has previously been associated with an increased risk of childhood asthma independently of thyroid hormone replacement [19]. Thus, as for Cluster 1, the cluster appears to provide more information on the maternal clinical context than on a specific pharmacological effect.

In contrast, Cluster 3 provides a potentially more informative signal regarding combined prenatal medication exposures. This profile was characterised by repeated exposure to antacids and systemic antibiotics throughout pregnancy, together with several other medication classes and a single dispensing of systemic corticosteroids during the first trimester, and was associated with a particularly high estimated risk of childhood asthma. Previous studies have reported associations between prenatal exposure to acid‐suppressing medications and childhood asthma and other allergic outcomes [20, 21, 22]. One proposed mechanism is that suppression of gastric acidity may impair the digestion of maternal dietary proteins, increasing exposure to intact food antigens and potentially promoting immune sensitisation. Prenatal antibiotic exposure has also been associated with childhood asthma in several observational studies [4, 23, 24]. Several mechanisms have been proposed, including disruption of maternal and/or infant gut microbiota, with potential consequences for immune maturation and regulation. However, these associations may also be affected by confounding, particularly by maternal infections and shared familial factors [25, 26, 27]. The combination of these exposures within Cluster 3 is of particular interest because it suggests that the joint occurrence and timing of several medication exposures may identify a higher‐risk context that would not necessarily emerge from analyses of individual medications. In particular, the concomitant exposure to medications that may influence immune development or sensitisation raises the hypothesis that combined prenatal exposures could contribute to childhood asthma risk. However, this interpretation remains exploratory. The cluster also included several other medications, including dermatological antifungals, gynaecological anti‐infectives and antiseptics, for which evidence of an association with childhood asthma is scarce or absent and for which no clear biological mechanism has been established. The extensive polypharmacy observed in this profile may therefore also reflect a greater maternal morbidity, symptom burden, propensity to seek healthcare, or prescribing patterns during pregnancy. These alternative explanations should be investigated in future studies.

Cluster 5 provides a complementary finding, as it was predominantly characterised by exposure to acid‐suppressing medications throughout pregnancy, together with medications for gastrointestinal functional disorders during the first trimester, and was associated with an increased risk of childhood asthma. The presence of this profile independently of the antibiotic exposure observed in Cluster 3 strengthens the rationale for further investigating the potential association between prenatal exposure to acid‐suppressing medications and childhood asthma. Nevertheless, as previously reported [20, 21, 22], confounding by indication remains an important concern, as maternal gastrointestinal disorders themselves may contribute to the observed association.

Finally, Cluster 4, characterised by repeated exposure to mineral supplements during the second and third trimesters, does not provide evidence for a specific medication‐related risk of childhood asthma. There is currently no established evidence suggesting that prenatal exposure to mineral supplementation increases the risk of childhood asthma. Instead, the characteristics of mothers in this cluster, including older age, higher employment rates and more frequent long‐term conditions and pregnancy‐related complications, suggest that this profile may represent a particular maternal healthcare and socioeconomic context. Although healthcare utilisation was not directly assessed in our study, differences in healthcare‐seeking behaviour and access to care could potentially influence both medication use during pregnancy and the likelihood of healthcare contacts and asthma treatment for the child. This interpretation further illustrates that medication exposure profiles may capture broader patterns of maternal health and healthcare utilisation rather than direct pharmacological effects alone.

Taken together, these findings highlight the added value of a profile‐based approach by capturing different contexts of prenatal medication use, including potential medication‐related effects, treatment indication and maternal morbidity. By considering multiple medication classes and their timing simultaneously, this approach provides a more comprehensive representation of real‐world prescribing patterns and may help generate hypotheses about potential risks that warrant further investigation. Although these profiles cannot currently be considered clinical risk scores, they may provide a useful basis for future research and, following external validation and causal assessment, could contribute to risk stratification in perinatal pharmacoepidemiology.

### 4.2 Methodological Considerations

The use of Bayesian profile regression in this study addresses the specific challenges posed by the analysis of multiple medication exposures during pregnancy. This semi‐supervised Bayesian clustering method has three decisive advantages over traditional approaches. First, simultaneous integration of exposures and outcomes: unlike traditional methods (*k*‐means, latent class analysis, hierarchical clustering) that operate exclusively on exposure variables, BPR simultaneously integrates exposure profiles and health outcomes during the clustering process. This innovation ensures that the profiles identified are not only homogeneous in terms of medication exposures, but also relevant to the risk of childhood asthma. Second, non‐parametric flexibility: the architecture based on the Dirichlet process avoids the need to set the number of clusters in advance, allowing the data structure to emerge naturally. Third, probabilistic quantification of uncertainty: the Bayesian approach using Markov Monte Carlo chains provides complete posterior distributions accompanied by credibility intervals. Previous applications of Bayesian profile regression in epidemiology have demonstrated its ability to identify clinically meaningful exposure profiles in contexts involving complex mixtures, including environmental exposures [28] and paediatric risk stratification [29]. Our study extends these applications to perinatal pharmacoepidemiology and highlights the potential of this approach as a tool for signal detection.

### 4.3 Strengths and Limitations

This study has several strengths. The large size of the POMME cohort provides sufficient statistical power to identify exposure profiles, even after excluding certain underrepresented medication subclasses. Moreover, the use of Bayesian profile regression enabled the joint analysis of a large number of correlated exposures, capturing real‐world patterns of co‐medication that are rarely addressed in perinatal pharmacoepidemiology.

However, certain limitations must be considered. First, even though asthma was identified using a previously validated algorithm based on medication dispensing data [12], this approach may be subject to misclassification, particularly in younger children where medications may be prescribed for transient respiratory conditions such as viral‐induced wheeze. We sought to limit this risk by restricting the outcome to children aged 4 years or older and requiring repeated dispensings of rescue or maintenance medications. The algorithm was also adapted to the French prescribing context, taking prescription duration into account. However, diagnostic codes or clinical records were not available in POMME to further validate the outcome. The observed asthma prevalence (15.2%) was consistent with estimates in the general French paediatric population (10%–16%), providing some reassurance regarding the validity of the outcome definition, although differences in prescribing practices may still have contributed to misclassification. Second, the exposure variables relied on medications that were prescribed and dispensed to the women, which are proxies for actual intake. Third, the pragmatic exposure threshold may have resulted in the exclusion of rare medication exposures, which could be considered in future studies using larger datasets. Fourth, the approach remains exploratory and lacks adjustment for potential confounding, particularly confounding by indication. Some identified medication profiles may reflect underlying maternal conditions that are themselves associated with childhood asthma, rather than effects of the medications. Potential confounders such as maternal age, calendar year of delivery, maternal asthma and other maternal characteristics could be considered. In addition, indication‐specific factors would need to be identified according to the signal under investigation. The identified associations should therefore be interpreted as hypothesis‐generating rather than as evidence of causal medication effects. Dedicated pharmacoepidemiological studies using appropriate causal frameworks and accounting for confounding are needed to further investigate these signals. Fifth, cluster formations did not incorporate potential confounding factors, which could influence the observed associations. Sixth, children without prenatal medication exposure were excluded because an unexposed profile would constitute a degenerate and uninformative category for the clustering approach, which aimed to characterise patterns of medication use among exposed pregnancies. However, the absence of an unexposed reference group limits the interpretation of the magnitude of the observed associations relative to no medication exposure. Future pharmacoepidemiological studies specifically designed to investigate the signals identified here should consider including an unexposed comparison group, where appropriate. Lastly, twins were treated as independent observations, without accounting for within‐pregnancy correlation. This may have influenced the observed associations, particularly as some medication exposure profiles included a higher proportion of twin pregnancies. Future studies should account for the clustering of children within pregnancies when investigating these signals.

### 4.4 Perspectives

The identification of prenatal medication exposure profiles associated with higher average asthma risk provides several avenues for future research. In particular, profiles characterised by co‐dispensing of antacids and antibiotics throughout pregnancy combined with a single dispensing of systemic corticosteroids in early pregnancy, and the profile marked by exposure to antibiotics, nasal medications and cough and cold medications concentrated in the third trimester, warrant further investigation using causal inference frameworks. Methodologically, the BPR offers a powerful and flexible framework for analysing multiple exposure situations in perinatal epidemiology. A recent extension of this approach, *PReMiuMlongi*, allows longitudinal or multivariate outcomes to be modelled in addition to covariates [30]. This development opens the possibility, in future work, of simultaneously examining changes in maternal exposures and children's health trajectories, thereby enriching the analysis of prenatal exposure profiles and their long‐term effects.

## 5 Conclusion

In this population‐based cohort study, we identified distinct prenatal medication exposure profiles associated with differing levels of childhood asthma risk using a semi‐supervised Bayesian approach. This exploratory analysis highlights combinations of medications and exposure windows that may not be captured by traditional single‐exposure methods and that warrant further investigation using causal inference frameworks. More broadly, Bayesian profile regression appears to be a valuable tool for signal detection in perinatal pharmacoepidemiology, with potential to inform future research on medication safety during pregnancy.

## Author Contributions

**Solène Russo:** formal analysis, writing‐original draft, visualisation. **Justine Bénévent:** methodology, writing‐review and editing, supervision. **Anna‐Belle Beau:** methodology, validation, writing‐original draft, supervision, guarantor. All authors critically revised the manuscript for important intellectual content and approved the final version.

## Funding

S.R. received financial support from INSERM and Univ Toulouse for the completion of a Master thesis during which the analyses for this study were conducted. No additional grant from any funding agency in the public, commercial or not‐for‐profit sectors.

## Ethics Statement

The study was approved by CNIL (Commission Nationale de l'Informatique et des Libertés), DR‐2013‐060. This study was performed on anonymised patient data. The mothers of the child included in POMME were informed of their inclusion and of the potential use of their anonymised data for research purposes. They can oppose the use of their data at any time.

## Conflicts of Interest

The authors declare no conflicts of interest.

## Supporting information


**Table S1:** Level 2 ATC code and name of the molecule used in the clustering.
**Table S2:** Classification of asthma medications: acute treatment and maintenance treatment.
**Table S3:** Main average probabilities of one dispensing and at least two dispensings, by trimester of pregnancy and medication subclass, with 95% credibility interval.
**Figure S4:** Transformed difference in average exposure probabilities (at least two dispensings) in the 6 high‐risk clusters. Only positive associations (values > 0), indicating higher prevalence of specific medication exposures within profiles, are interpreted as the negative values indicate a lower prevalence of specific medication exposures in a profile.

## Data Availability

The data relevant to this study cannot be shared publicly as it contains sensitive health data, including data about children and potentially identifying information due to small sizes of some subpopulations. Additionally, the Commission Nationale de l'Informatique et des Libertés (CNIL) does not authorise the public sharing of the POMME data. Data access requires the submission of a research project according to a strict protocol to the cohort investigators and a request for individual access to the secure computing server. For more information, contact the Data Protection Officer (dpo@chu-toulouse.fr).

## References

[1] I. Lacroix , C. Damase‐Michel , M. Lapeyre‐Mestre , and J. L. Montastruc , “Prescription of Drugs During Pregnancy in France,” Lancet 356 (2000): 1735–1736, 10.1016/s0140-6736(00)03209-8. 11095263

[2] M.‐C. Delmas , B. Leynaert , L. Com‐Ruelle , I. Annesi‐Maesano , and C. Fuhrman , “Asthme: Prévalence et Impact sur la vie Quotidienne. Analyse des Données de L'enquête Décennale Santé 2003 de L'INSEE,” 2008, http://www.invs.sante.fr/publications/2008/asthme/RAPP_SCI_Asthme%202003_Web.pdf.

[3] E. Hak , B. Mulder , C. C. M. Schuiling‐Veninga , T. W. de Vries , and S. S. Jick , “Use of Acid‐Suppressive Drugs in Pregnancy and the Risk of Childhood Asthma: Bidirectional Crossover Study Using the General Practice Research Database,” Drug Safety 36 (2013): 1097–1104, 10.1007/s40264-013-0093-z. PMC3824882 24018582

[4] C. S. Uldbjerg , J. E. Miller , D. Burgner , L. H. Pedersen , and B. H. Bech , “Antibiotic Exposure During Pregnancy and Childhood Asthma: A National Birth Cohort Study Investigating Timing of Exposure and Mode of Delivery,” Archives of Disease in Childhood 106 (2021): 888–894, 10.1136/archdischild-2020-319659. 33563603

[5] R. Baron , M. Taye , I. B. der Vaart , et al., “The Relationship of Prenatal Antibiotic Exposure and Infant Antibiotic Administration With Childhood Allergies: A Systematic Review,” BMC Pediatrics 20 (2020): 312, 10.1186/s12887-020-02042-8. PMC7320596 32593308

[6] Y. Noh , H. E. Jeong , A. Choi , et al., “Prenatal and Infant Exposure to Acid‐Suppressive Medications and Risk of Allergic Diseases in Children,” JAMA Pediatrics 177 (2023): 267–277, 10.1001/jamapediatrics.2022.5193. PMC9857801 36622684

[7] A. Subramanian , A. Azcoaga‐Lorenzo , A. Anand , et al., “Polypharmacy During Pregnancy and Associated Risk Factors: A Retrospective Analysis of 577 Medication Exposures Among 1.5 Million Pregnancies in the UK, 2000‐2019,” BMC Medicine 21 (2023): 21, 10.1186/s12916-022-02722-5. PMC9843951 36647047

[8] J. Benevent , C. Hurault‐Delarue , M. Araujo , J.‐L. Montastruc , I. Lacroix , and C. Damase‐Michel , “POMME: The New Cohort to Evaluate Long‐Term Effects After Prenatal Medicine Exposure,” Drug Safety 42 (2019): 45–54, 10.1007/s40264-018-0712-9. 30121741

[9] EUROCAT , “EUROCAT Guide 1.4: Instruction for the Registration of Congenital Anomalies,” EUROCAT Central Registry, University of Ulster, 2013, accessed December 8, 2020, https://eu‐rd‐platform.jrc.ec.europa.eu/sites/default/files/JRC‐EUROCAT‐Full‐Guide‐1.4‐version‐15‐Nov‐2019.pdf.

[10] “ATCDDD – ATC/DDD Index,” accessed December 12, 2025, https://atcddd.fhi.no/atc_ddd_index/.

[11] Committee Opinion No 700 , “Methods for Estimating the due Date,” Obstetrics & Gynecology 129 (2017): e150–e154. 10.1097/AOG.0000000000002046 28426621

[12] S. Rubak , A. Høst , L. B. Christensen , M. S. Langfrits , and R. W. Thomsen , “Validity of Asthma Diagnoses and Patterns of Anti‐Asthmatic Drug Use in a Cohort of 2053 Danish Children,” Health Science Reports 1 (2018): e77, 10.1002/hsr2.77. PMC6266370 30623100

[13] S. Webmaster , Recommandations Pour la Prise en Charge et le Suivi des Patients Asthmatiques Adultes (Société de Pneumologie de Langue Française, 2022), accessed July 5, 2024, https://splf.fr/recommandations‐pour‐la‐prise‐en‐charge‐et‐le‐suivi‐des‐patients‐asthmatiques‐adultes‐2021/.

[14] “2024 GINA Main Report. Global Initiative for Asthma – GINA,” accessed July 5, 2024, https://ginasthma.org/2024‐report/.

[15] L. Rasmussen , B. Wettermark , D. Steinke , and A. Pottegård , “Core Concepts in Pharmacoepidemiology: Measures of Drug Utilization Based on Individual‐Level Drug Dispensing Data,” Pharmacoepidemiology and Drug Safety 31 (2022): 1015–1026, 10.1002/pds.5490. PMC9545237 35819240

[16] J. Molitor , M. Papathomas , M. Jerrett , and S. Richardson , “Bayesian Profile Regression With an Application to the National Survey of Children's Health,” Biostatistics 11 (2010): 484–498, 10.1093/biostatistics/kxq013. 20350957

[17] S. Liverani , D. I. Hastie , L. Azizi , M. Papathomas , and S. Richardson , “PReMiuM: An R Package for Profile Regression Mixture Models Using Dirichlet Processes,” Journal of Statistical Software 64 (2015): 1–30, 10.18637/jss.v064.i07. PMC4905523 27307779

[18] A. J. Roff , J. L. Robinson , S. J. Hammond , et al., “Maternal Asthma During Pregnancy and Risks of Allergy and Asthma in Progeny: A Systematic Review and Meta‐Analysis,” BJOG 132 (2025): 1045–1055, 10.1111/1471-0528.17900. PMC12137787 38973390

[19] X. Liu , S. L. Andersen , J. Olsen , et al., “Maternal Hypothyroidism in the Perinatal Period and Childhood Asthma in the Offspring,” Allergy 73 (2018): 932–939, 10.1111/all.13365. 29159833

[20] E. Dehlink , E. Yen , A. M. Leichtner , E. J. Hait , and E. Fiebiger , “First Evidence of a Possible Association Between Gastric Acid Suppression During Pregnancy and Childhood Asthma: A Population‐Based Register Study,” Clinical and Experimental Allergy 39 (2009): 246–253, 10.1111/j.1365-2222.2008.03125.x. 19134022

[21] B. Mulder , C. C. M. Schuiling‐Veninga , H. J. Bos , T. W. De Vries , S. S. Jick , and E. Hak , “Prenatal Exposure to Acid‐Suppressive Drugs and the Risk of Allergic Diseases in the Offspring: A Cohort Study,” Clinical and Experimental Allergy 44 (2014): 261–269, 10.1111/cea.12227. 24164287

[22] A. B. T. Andersen , R. Erichsen , D. K. Farkas , F. Mehnert , V. Ehrenstein , and H. T. Sørensen , “Prenatal Exposure to Acid‐Suppressive Drugs and the Risk of Childhood Asthma: A Population‐Based Danish Cohort Study,” Alimentary Pharmacology & Therapeutics 35 (2012): 1190–1198, 10.1111/j.1365-2036.2012.05073.x. 22443179

[23] S. Yoshida , K. Ide , M. Takeuchi , and K. Kawakami , “Prenatal and Early‐Life Antibiotic Use and Risk of Childhood Asthma: A Retrospective Cohort Study,” Pediatric Allergy and Immunology 29 (2018): 490–495, 10.1111/pai.12902. 29604125

[24] K. N. Turi , T. Gebretsadik , T. Ding , et al., “Dose, Timing, and Spectrum of Prenatal Antibiotic Exposure and Risk of Childhood Asthma,” Clinical Infectious Diseases 72 (2021): 455–462, 10.1093/cid/ciaa085. PMC7850553 31994697

[25] A. K. Örtqvist , C. Lundholm , H. Kieler , et al., “Antibiotics in Fetal and Early Life and Subsequent Childhood Asthma: Nationwide Population Based Study With Sibling Analysis,” BMJ 349 (2014): g6979, 10.1136/bmj.g6979. PMC4247260 25432937

[26] K. Loewen , B. Monchka , S. M. Mahmud , G. ’t. Jong , and M. B. Azad , “Prenatal Antibiotic Exposure and Childhood Asthma: A Population‐Based Study,” European Respiratory Journal 52, no. 1 (2018): 1702070–1702080, 10.1183/13993003.02070-2017. 29678946

[27] N. C. Momen and X. Liu , “Maternal Antibiotic Use During Pregnancy and Asthma in Children: Population‐Based Cohort Study and Sibling Design,” European Respiratory Journal 57 (2021): 2000937, 10.1183/13993003.00937-2020. 33446576

[28] C. Giampiccolo , A. Amadou , T. Coudon , et al., “Multi‐Pollutant Exposure Profiles Associated With Breast Cancer Risk: A Bayesian Profile Regression Analysis in the French E3N Cohort,” Environment International 190 (2024): 108943, 10.1016/j.envint.2024.108943. 39137687

[29] P. Gilholm , S. Raman , A. Irwin , et al., “Identification of Distinct Clinical Profiles of Sepsis Risk in Paediatric Emergency Department Patients Using Bayesian Profile Regression,” BMJ Paediatrics Open 9 (2025): e003100, 10.1136/bmjpo-2024-003100. PMC11906992 40074245

[30] A. Rouanet , R. Johnson , M. Strauss , et al., “Bayesian Profile Regression for Clustering Analysis Involving a Longitudinal Response and Explanatory Variables,” Methodology (Gott) 73 (2024): 314–339, 10.1093/jrsssc/qlad097. PMC7615733 38577633

