# Supplementary material for: Identification of Risk‐Related Prenatal Medication Exposure Profiles for Childhood Asthma Using the Bayesian Profile Regression: A Cohort Study Using the POMME Cohort
Source: Pharmacoepidemiol Drug Saf. 2026 Oct 19;35(11):e70509. doi: 10.1002/pds.70509 (PMC13680107; doi:10.1002/pds.70509)
Supplement: Supplementary file 1 [file PDS-35-e70509-s001.docx]

**Supplementary data**

**journal:** PDS

**Title:** Identification of risk-related prenatal medication exposure profiles for childhood asthma using the Bayesian profile regression: a cohort study using the POMME cohort.

**Authors**

**Solène Russo^1^, Justine Bénévent^2^, Anna-Belle Beau^2*^**

**Authors affiliation**

1. CERPOP, Inserm U1295, Toulouse (France)

2. CERPOP, Inserm U1295, Université de Toulouse, Service de Pharmacologie Clinique, CHU de Toulouse, Toulouse (France)

*** Corresponding author**

Anna-Belle Beau

Toulouse University Hospital, CERPOP-SPHERE Team, Inserm UMR 1295

37 allées Jules Guesde Bat C floor 3, 31000 Toulouse, France

Phone: +33 5 61 14 59 43

Anna-Belle Beau ORCID: 0000-0003-4631-100X

**Table S1**: Level 2 ATC code and name of the molecule used in the clustering

| **ATC code** | **Name of the molecule** |
| --- | --- |
| A01 | Stomatological preparations |
| A02 | Medicines for acidity-related disorders |
| A03 ***** | Medicines for gastrointestinal functional disorders |
| A04 | Antiemetics and anti-nausea medicines |
| A06 | Laxatives |
| A07 | Antidiarrhoeals, anti-inflammatory/anti-infective agents for the intestine |
| A10 | Medicines used in diabetology |
| A12 | Mineral supplements |
| B01 ***** | Antithrombotic agents |
| C08 | Calcium channel blockers |
| D01 | Antifungals for dermatological use |
| D02 | Emollients and protectors |
| D06 | Antibiotics and chemotherapeutics for dermatological use |
| D07 | Corticosteroids for dermatological use, preparations |
| D08 | Antiseptics and disinfectants |
| D10 | Anti-acne preparations |
| G01 | Gynecological anti-infectives and antiseptics |
| G02 | Other gynecological medicines |
| G03 | Sex hormones and modulators of the genital system |
| H02 | Corticosteroids for systemic use |
| H03 | Thyroid therapies |
| J01 | Antibacterials for systemic use |
| J05 | Antivirals for systemic use |
| J06 | Immunological sera and immunoglobulins |
| J07 | Vaccines |
| M01 | Anti-inflammatories and antirheumatics |
| M02 | Topical treatments for joint and muscle conditions |
| N02 ***** | Analgesics |
| N05 | Psycholeptics |
| P01 | Antiparasitics |
| R01 | Nasal preparations |
| R03 | Medicines for obstructive airway diseases |
| R05 | Medicines for coughs and colds |
| R06 | Systemic antihistamines |
| S01 | Ophthalmologicals |
| S02 | Otologicals |

*Excluding paracetamol (N02BE01), phloroglucinol (A03AX12) and heparin sodium (B01AB01)

| **Type** | **Dispensing criteria** | **ATC code** | **Name of active substance** |
| --- | --- | --- | --- |
| **intermittent asthma**  (Fast-acting bronchodilators) | At least 3 dispensings | R03AC02  R03AC03  R03AC04  R03AC08  R03BB01 | Salbutamol  Terbutaline  Fenoterol  Pirbuterol  Ipratropium |
| **persistent asthma**  (Continuous treatment) | At least 3 consecutive dispensings, with a maximum interval of 42 days between each dispensing | R03AC13  R03AC12  R03AC18  R03AC19  R03DC03  R03BB04  R03BA  R03DA  R03AK  R03DX | Formoterol  Salmeterol  Indocaterol  Olodaterol  Montelukast  Tiotropium  Inhaled glucocorticoids  Xanthines  Combinations of beta-2 agonists + inhaled corticosteroids  Autres médicaments pour troubles obstructifs voies respiratoires |

**Table S2**: Classification of asthma medications: acute treatment and maintenance treatment

**Table S3**: Main average probabilities of one dispensing and at least two dispensings, by trimester of pregnancy and medication subclass, with 95% credibility interval.

|  | Cluster 1  n = 364 | Cluster 2  n = 188 | Cluster 3  n = 483 | Cluster 4  n = 99 | Cluster 5  n = 677 | Cluster 6  n = 1 019 | Reference group  n =12 653 |
| --- | --- | --- | --- | --- | --- | --- | --- |
| Estimated risk of asthma (%) | 27.4% | 26.2% | 25.3% | 25.0% | 18.4% | 18.2% | 13.8% |
| **First trimester of pregnancy** | | | | | | | |
| **A02: Medicines for acid-related disorders** | | | | | | | |
| 1 dispensing  At least two dispensings | 12.3%  [9.6 – 15.1]  7.3%  [5.2 – 9.6] | 16.5%  [11.8 – 24.8]  11.3%  [7.6 – 15.9] | 21.4%  [18.1 – 24.8]  17.5%  [14.6 – 20.6] | 9.3%  [4.8 – 15.1]  4.8%  [1.9 – 9.2] | 22.4%  [19.6 – 25.1]  13.7%  [11.5 – 16.0] | 7.6%  [6.3 – 9.0]  2.4%  [1.8 – 3.2] | 7.1%  [6.6 – 7.5]  2.1%  [1.8 – 2.3] |
| **A03: Medicines for gastro intestinal functional disorders (excluding phloroglucinol)** | | | | | | | |
| 1 dispensing  At least two dispensings | 19.6%  [16.3 – 23.3]  6.4%  [4.6 – 85] | 16.6%  [12.0– 21.6]  9.9%  [6.3 – 14.3] | 29.2%  [25.6 – 32.6]  17.4%  [14.5 – 20.5] | 23.1%  [15.9– 31.0]  2.3%  [0.9 – 5.2] | 26.4%  [23.6 – 29.1]  15.1%  [12.8 – 17.5] | 15.0%  [13.3– 16.9]  4.4%  [3.5 – 5.5] | 14.8%  [14.2 – 15.4]  3.5%  [3.2 – 3.8] |
| **A12: Mineral supplements** | | | | | | | |
| 1 dispensing  At least two dispensings | 2.6%  [1.6 – 3.9]  0.8%  [0.3 – 1.7] | 5.3%  [2.5 -8.9]  1.6%  [0.5 – 3.6] | 7.1%  [5.1 – 9.2]  1.0%  [0.5 – 1.8] | 30.1%  [21.4 – 39.4]  17.6%  [11.5 – 25.1] | 5.5%  [4.1 – 6.9]  1.1%  [0.6 – 1.8] | 2.3%  [1.6 – 3.1]  0.3%  [0.2 – 0.7] | 2.2%  [2.0 – 2.4]  0.3%  [0.3 – 0.4] |
| **D01: Antifungals for dermatological use** | | | | | | | |
| 1 dispensing  At least two dispensings | 9.6%  [7.3 – 18.2]  3.2%  [2.0 – 4.8] | 16.0%  [11.3 -21.3]  5.6%  [3.1 – 8.9] | 22.2%  [18.9 – 25.7]  6.4%  [7.2 – 11.8] | 12.1%  [7.4 – 18.2]  5.9%  [2.6 – 10.8] | 7.3%  [5.9 – 8.9]  3.2%  [2.2 – 4.3] | 4.2%  [3.4 – 5.3]  1.6%  [1.1 – 2.3] | 6.5%  [6.2 – 6.9]  1.8%  [1.6 – 2.0] |
| **G01: Gynecological anti-infectives and antiseptics** | | | | | | | |
| 1 dispensing  At least two dispensings | 7.2%  [5.2 – 9.6]  2.3%  [1.3 – 3.6] | 13.2%  [9.0 -18.1]  4.0%  [1.8 – 7.1] | 20.6%  [17.5 – 23.8]  7.3%  [5.3 – 9.5] | 12.11%  [7.3 – 17.8]  1.9%  [0.5 – 4.4] | 13.2%  [9.0 – 18.1]  1.1%  [0.7 – 1.7] | 6.7%  [5.4– 8.3]  0.4%  [0.3 – 0.7] | 3.0%  [2.3 – 3.9]  0.9%  [0.8 – 1.1] |
| **H02: Corticosteroids for systemic use** | | | | | | | |
| 1 dispensing  At least two dispensings | 14.3%  [11.3 – 17.6]  3.8%  [2.3 – 5.7] | 11.0%  [7.1 -15.5]  3.9%  [1.7 – 6.8] | 17.7%  [14.7 – 20.7]  4.2%  [2.7 – 5.9] | 7.3%  [3.4 – 12.4]  5.5%  [1.2 – 6.9] | 6.8%  [5.6 – 8.3]  1.3%  [0.8– 2.1] | 4.3%  [3.3 – 5.3]  0.4%  [0.3 – 0.6] | 4.6%  [4.3 – 4.9]  0.9%  [0.7 – 1.1] |
|  |  |  |  |  |  |  |  |
| **H03: Thyroid therapies** | | | | | | | |
| 1 dispensing  At least two dispensings | 1.0%  [0.4 – 2.1]  1.1%  [0.5 – 2.0] | 13.3%  [9.2 – 18.2]  78.5%  [72.9 – 83.6] | 0.9%  [0.4 – 1.8]  1.0%  [0.5 – 1.9] | 1.0%  [0.1 – 3.2]  1.4%  [0.2 – 3.8] | 0.7%  [0.3 – 1.4]  0.3%  [0.1 – 0.6] | 0.2%  [0.1 – 0.5]  0.4%  [0.1 – 0.7] | 1.6%  [1.4 – 1.8]  5.4%  [5.2 – 5.6] |
| **J01: Antibacterial for systemic use** | | | | | | | |
| 1 dispensing  At least two dispensings | 22.7%  [19.2 – 26.5]  11.5%  [8.9 – 14.6] | 28.5%  [22.6 – 34.7]  13.6%  [9.4 – 18.6] | 31.8%  [28.0 – 35.6]  19.7%  [16.5 – 23.3] | 23.7%  [16.7 – 31.5]  4.6%  [2.0 – 8.8] | 16.9%  [14.7 – 19.2]  5.2%  [4.1 – 6.6] | 13.1%  [11.5 – 15.0]  3.5%  [2.7 – 4.5] | 14.1%  [13.5 – 14.6]  4.1%  [3.8 – 4.4] |
| **M01: Anti-inflammatories and antirheumatics** | | | | | | | |
| 1 dispensing  At least two dispensings | 9.6%  [7.5 – 12.2]  2.2%  [1.4 – 3.5] | 17.6%  [12.5 -23.1]  4.6%  [2.4 – 7.9] | 23.25%  [20.0 – 26.7]  8.4%  [6.3 – 10.8] | 8.1%  [4.1 – 13.4]  2.6%  [0.7 – 5.7] | 14.2%  [12.1– 16.4]  3.6%  [2.5 – 5.0] | 6.5%  [5.4 – 7.8]  0.7%  [0.5 – 1.2] | 7.3%  [6.9 – 7.7]  1.1%  [0.9 – 1.2] |
| **R03: Medicines for obstructive airway diseases** | | | | | | | |
| 1 dispensing  At least two dispensings | 18.1%  [14.5 – 21.9]  21.4%  [17.8 – 25.3] | 9.3%  [5.9 – 13.7]  7.3%  [4.4 – 10.9] | 9.2%  [7.0 – 11.6]  6.0%  [4.4 – 8.0] | 3.6%  [1.4 – 7.2]  2.2%  [0.6 – 5.1] | 2.9%  [2.2 – 3.7]  1.1%  [0.7 – 1.6] | 2.9%  [2.2 – 3.8]  1.3%  [0.9 – 1.8] | 2.5%  [2.2 – 2.7]  0.9%  [0.8 – 1.1] |
| **R05: Medicines for coughs and colds** | | | | | | | |
| 1 dispensing  At least two dispensings | 17.4%  [14.2 – 20.9]  3.8%  [2.2 – 5.9] | 17.5%  [12.6 -23.0]  6.2%  [3.4 – 9.8] | 21.7%  [18.4 – 25.1]  7.1%  [5.0 – 9.4] | 12.1%  [7.3 – 18.3]  3.9%  [1.3 – 8.1] | 10.3%  [8.7 – 12.0]  1.9%  [1.2 – 2.7] | 7.7%  [6.4 – 9.1]  1.0%  [0.6 – 1.5] | 8.1%  [7.7 – 8.5]  1.2%  [1.0 – 1.4] |
| **R06: Systemic antihistamines** | | | | | | | |
| 1 dispensing  At least two dispensings | 22.1%  [18.1 -26.1]  17.8%  [14.3 – 21.5] | 9.4%  [5.9 – 13.7]  8.4%  [5.2 – 12.3] | 16.9%  [13.9 – 20.1]  5.8%  [4.1 – 7.7] | 5.8%  [2.7 – 10.6]  1.5%  [0.3 – 4.0] | 6.2%  [5.0 – 7.6]  2.2%  [1.4 – 3.1] | 4.5%  [3.6 – 5.5]  1.2%  [0.8 – 1.8] | 4.7%  [4.3 – 5.0]  1.1%  [0.9 – 1.2] |
| **Second trimester of pregnancy** | | | | | | | |
| **A02: Medicines for acid-related disorders** | | | | | | | |
| 1 dispensing  At least two dispensings | 14.1%  [11.3 – 17.4]  10.5%  [8.0 – 13.3] | 17.3%  [12.2 – 22.9]  16.1%  [11.5 – 21.4] | 22.63%  [19.2 – 26.2]  17.6%  [14.6 – 20.7] | 8.1%  [4.2 – 13.5]  4.5%  [1.5 – 9.2] | 18.5%  [15.9 – 21.3]  13.8%  [11.5 – 16.2] | 10.8%  [9.3 – 12.5]  3.1%  [2.4 – 4.0] | 9.2%  [8.8 – 9.7]  3.0%  [2.7 – 3.3] |
| **A12: Mineral supplements** | | | | | | | |
| 1 dispensing  At least two dispensings | 4.5%  [2.9 – 6.4]  1.3%  [0.6 – 2.3] | 2.7%  [1.3 – 5.1]  2.4%  [0.9 – 4.8] | 8.3%  [6.3 – 10.5]  2.0%  [1.1 – 3.2] | 32.1%  [21.4 – 43.4]  42.1%  [32.6 – 51.7] | 5.5%  [4.3 – 6.9]  1.7%  [1.1 – 2.6] | 3.3%  [2.5 – 4.3]  0.7%  [0.4 – 1.2] | 3.5%  [3.2 – 3.8]  0.8%  [0.7 – 0.9] |
| **D01: Antifungals for dermatological use** | | | | | | | |
| 1 dispensing  At least two dispensings | 10.6%  [8.2 – 13.2]  5.0%  [3.4 – 6.9] | 20.6%  [15.5– 26.1]  5.3%  [2.6 – 8.7] | 24.9%  [21.1 – 28.6]  14.0%  [11.4 – 16.9] | 17.3%  [11.3 – 24.7]  3.3%  [1.1 – 7.1] | 7.7%  [6.1– 9.4]  3.3%  [2.4 – 4.6] | 6.1%  [5.0 – 7.4]  2.1%  [1.5 – 2.9] | 9.3%  [8.9– 9.6]  3.0%  [2.8 – 3.3] |
| **H03: Thyroid therapies** | | | | | | | |
| 1 dispensing  At least two dispensings | 0.9%  [0.3 – 2.1]  1.0%  [0.4 – 1.9] | 10.9%  [6.9 – 15.4]  86.0%  [81.2 – 90.2] | 0.7%  [0.2 – 1.5]  0.3%  [0.1 – 0.9] | 0.8%  [0.1 – 2.9]  0.8%  [0.1 – 2.8] | 0.6%  [0.3 – 1.2]  0.3%  [0.1 – 0.6] | 0.2%  [0.1 – 0.5]  0.2%  [0.0 – 0.4] | 1.3%  [1.1 – 1.5]  6.0%  [5.8 – 6.2] |
| **J01: Antibacterial for systemic use** | | | | | | | |
| 1 dispensing  At least two dispensings | 27.4%  [23.5 – 31.4]  11.6%  [9.0 – 14.7] | 21.0%  [16.1 – 26.3]  8.9%  [5.6 - 13.0] | 29.3%  [25.6 – 33.0]  19.0%  [15.8 – 22.3] | 20.8%  [14.5 – 27.9]  8.2%  [4.5 – 13.3] | 14.7%  [12.7 – 16.9]  6.3%  [5.0 – 7.8] | 16.6%  [14.6 – 18.6]  5.5%  [4.4 – 6.7] | 13.8%  [13.2 - 14.4]  4.4%  [4.0 – 4.7] |
| **R01: Nasal preparations** | | | | | | | |
| 1 dispensing  At least two dispensings | 29.5%  [25.6 – 33.4]  13.7%  [10.7 – 16.9] | 24.1%  [18.6 – 29.8]  7.7%  [4.5 – 11.5] | 27.0%  [23.5 – 30.5]  13.0%  [10.5 – 15.9] | 18.1%  [11.8 – 25.3]  2.9%  [1.0 – 6.4] | 13.2%  [11.2– 15.2]  1.8%  [1.3 – 2.6] | 15.6%  [13.7 – 17.6]  1.8%  [1.3 – 2.5] | 11.8%  [11.3– 12.3]  1.5%  [1.3 – 1.7] |
| **R03: Medicines for obstructive airway diseases** | | | | | | | |
| 1 dispensing  At least two dispensings | 17.2%  [13.6 – 21.0]  27.2%  [23.0 – 31.6] | 5.1%  [2.5 -8.5]  5.7%  [3.1 – 9.2] | 11.6%  [9.1 – 14.2]  5.8%  [4.2 – 7.7] | 7.0%  [3.5 – 11.9]  3.4%  [1.2 – 6.9] | 3.4%  [2.5 – 4.5]  1.2%  [0.8 – 1.9] | 4.8%  [3.7 – 6.1]  1.9%  [1.3 – 2.6] | 3.0%  [2.7 – 3.3]  1.1%  [0.9 – 1.3] |
| **R05: Medicines for coughs and colds** | | | | | | | |
| 1 dispensing  At least two dispensings | 16.4%  [13.4 – 19.6]  3.6%  [2.3 – 5.3] | 19.3%  [14.4 -24.5]  2.8%  [1.0 – 5.5] | 21.8%  [18.5 – 25.3]  6.5%  [4.7 – 8.7] | 14.6%  [8.9 – 21.4]  1.9%  [0.6 – 4.6] | 10.7%  [9.3 – 12.6]  1.4%  [0.9 – 2.2] | 11.6%  [10.0 – 13.2]  1.7%  [1.2 – 2.5] | 8.4%  [8.0 – 8.8]  1.2%  [1.0 – 1.3] |
| **R06: Systemic antihistamines** | | | | | | | |
| 1 dispensing  At least two dispensings | 17.9%  [14.6 – 21.7]  21.3%  [17.3 – 25.4] | 10.3%  [6.8 – 14.6]  7.5%  [4.4 – 11.1] | 11.0%  [8.8 – 13.7]  3.1%  [2.0 – 4.5] | 3.9%  [1.5 – 7.6]  2.3%  [0.7 – 5.6] | 3.5%  [2.7 – 4.5]  1.4%  [0.9 – 2.2] | 3.5%  [2.6 – 4.5]  1.1%  [0.8 – 1.6] | 3.5%  [3.2 – 3.8]  0.8%  [0.7 – 1.0] |
| **Third trimester of pregnancy** | | | | | | | |
| **A02: Medicines for acid-related disorders** | | | | | | | |
| 1 dispensing  At least two dispensings | 15.7%  [12.7 - 22.9]  19.3%  [16.1 – 22.9] | 17.5%  [12.8 - 22.9]  22.2%  [17.1 – 28.0] | 21.1%  [18.1 - 24.4]  27.0%  [23.2 – 30.7] | 13.9%  [8.5 - 20.1]  11.2%  [6.1 – 17.7] | 19.5%  [17.2 - 22.0]  22.2%  [19.4 – 25.1] | 17.8%  [15.9 - 19.9]  13.2%  [11.4 – 15.0] | 14.6%  [14.0 - 15.2]  9.1%  [8.6 – 9.5] |
| **A12: Mineral supplements** | | | | | | | |
| 1 dispensing  At least two dispensings | 4.7%  [3.1 – 6.7]  1.7%  [0.9 – 2.8] | 6.4%  [3.6 – 10.1]  4.9%  [2.5 – 8.0] | 5.6%  [4.0 – 7.5]  2.7%  [1.6 – 4.0] | 23.1%  [13.7 – 32.9]  39.7%  [30.0 – 49.3] | 4.0%  [3.0 – 5.3]  1.3%  [0.8 – 2.0] | 3.9%  [3.0 – 4.9]  1.0%  [0.6 – 1.6] | 3.4%  [3.1 – 3.7]  1.1%  [0.9 – 1.3] |
| **D01: Antifungals for dermatological use** | | | | | | | |
| 1 dispensing  At least two dispensings | 12.6%  [10.0 – 15.6]  4.3%  [2.9 – 6.1] | 12.9%  [8.8– 17.8]  8.0%  [4.7 – 11.9] | 24.5%  [21.3 – 28.1]  16.2%  [13.3 – 19.2] | 16.2%  [10.7 – 22.8]  3.9%  [1.3 – 8.2] | 9.2%  [7.5 – 11.1]  2.8%  [2.2 – 3.7] | 10.7%  [9.1 – 12.3]  3.7%  [2.8 – 4.8] | 10.0%  [9.5– 10.4]  3.6%  [3.3 – 3.9] |
| **G01:** **Gynecological anti-infectives and antiseptics** | | | | | | | |
| 1 dispensing  At least two dispensings | 12.5%  [9.8 – 15.6]  3.8%  [2.6 – 5.5] | 17.0%  [12.4 – 22.1]  7.0%  [3.9 – 10.9] | 23.4%  [20.1 – 26.9]  16.0%  [13.1 – 19.1] | 14.6%  [9.3 – 21.1]  8.7%  [4.7 – 14.0] | 11.0%  [9.3 – 13.0]  2.9%  [2.2 – 3.9] | 11.5%  [9.9 – 13.2]  3.0%  [2.3 – 3.9] | 12.5%  [12.0 – 13.0]  3.7%  [3.4 – 4.0] |
| **H03: Thyroid therapies dispensed** | | | | | | | |
| 1 dispensing  At least two dispensings | 0.8%  [0.2 – 1.8]  1.4%  [0.7 – 2.5] | 8.0%  [4.9 – 11.9]  88.5%  [84.1 – 92.3] | 0.3%  [0.1 – 0.9]  0.5%  [0.1 – 1.1] | 1.5%  [0.2 – 4.1]  0.9%  [0.1 – 2.9] | 0.4%  [0.2 – 0.8]  0.4%  [0.2 – 0.8] | 0.4%  [0.2 – 0.8]  0.6%  [0.3 – 1.2] | 1.1%  [1.0 – 1.3]  6.4%  [6.2 – 6.6] |
| **J01: Antibacterial for systemic use** | | | | | | | |
| 1 dispensing  At least two dispensings | 21.5%  [18.0 – 25.2]  10.5%  [8.1 – 13.4] | 22.9%  [17.8 – 28.6]  9.5%  [6.1 – 13.5] | 28.5%  [25.1 – 32.1]  20.1%  [16.9 – 23.4] | 12.4%  [7.5 – 18.7]  9.3%  [5.1 – 14.7] | 14.1%  [12.1 – 16.1]  4.3%  [3.3 – 5.6] | 37.1%  [34.5 – 39.8]  15.4%  [13.3 – 17.6] | 11.7%  [11.2 – 12.3]  3.9%  [3.6 – 4.2] |
| **R01: Nasal preparations** |  |  |  |  |  |  |  |
| 1 dispensing  At least two dispensings | 22.1%  [18.6 – 26.0]  7.5%  [5.4 – 10.1] | 20.8%  [15.9 – 26.4]  7.1%  [4.1 – 10.9] | 27.3%  [23.7 – 30.9]  9.9%  [7.6 – 12.4] | 13.3%  [8.2– 19.3]  1.4%  [0.4 – 3.5] | 9.2%  [7.7 – 11.1]  1.8%  [1.2 – 2.5] | 39.5%  [36.6 – 42.4]  9.3%  [7.8 – 10.9] | 7.4%  [7.0 – 7.9]  0.9%  [0.8 – 1.1] |
| **R03: Medicines for obstructive airway diseases** | | | | | | | |
| 1 dispensing  At least two dispensings | 17.3%  [13.9 – 21.0]  22.8%  [18.9 – 26.8] | 6.2%  [3.3 – 9.9]  8.4%  [5.2 – 12.2] | 12.4%  [9.9 – 15.1]  7.9%  [5.9 – 10.2] | 5.9%  [2.6 – 10.6]  2.6%  [0.7 – 6.0] | 2.8%  [2.1 – 3.8]  0.9%  [0.6 – 1.3] | 15.4%  [13.5 – 17.5]  6.1%  [4.9 – 7.5] | 2.4%  [2.1 – 2.6]  0.9%  [0.8 – 1.1] |
| **R05: Medicines for coughs and colds** | | | | | | | |
| 1 dispensing  At least two dispensings | 10.7%  [7.9– 12.8]  1.7%  [1.1 – 2.8] | 14.5%  [10.2 – 19.3]  5.1%  [2.6 – 8.4] | 22.6%  [19.5 – 26.1]  7.0%  [5.1 – 9.1] | 7.6%  [3.9 – 12.8]  1.1%  [0.3 – 2.9] | 7.0%  [5.6 – 8.6]  1.2%  [0.8 – 1.8] | 35.5%  [32.6 – 38.3]  7.3%  [5.9 – 8.8] | 5.3%  [4.9 – 5.6]  0.7%  [0.5 – 0.8] |
| **R06: Systemic antihistamines** | | | | | | | |
| 1 dispensing  At least two dispensings | 12.3%  [9.5 – 18.5]  20.7%  [16.8 – 24.7] | 13.7%  [9.8 – 18.5]  3.0%  [1.1 – 5.7] | 11.5%  [9.1 – 14.4]  3.4%  [2.1 – 4.8] | 6.4%  [3.0 – 11.0]  1.6%  [0.4 – 4.1] | 3.9%  [2.9 – 5.2]  1.1%  [0.6 – 1.7] | 7.9%  [6.5 – 9.4]  2.6%  [1.8 – 3.5] | 2.7%  [2.4 – 3.0]  0.7%  [0.6 – 0.9] |

**Figure S4**. Transformed difference in average exposure probabilities (at least two dispensings) in the 6 high-risk clusters. Only positive associations (values >0), indicating higher prevalence of specific medication exposures within profiles, are interpreted as the negative values indicate a lower prevalence of specific medication exposures in a profile.
